# Expression of Cytokines and Chemokines as Predictors of Stroke Outcomes in Acute Ischemic Stroke

**DOI:** 10.3389/fneur.2019.01391

**Published:** 2020-01-15

**Authors:** Sarah R. Martha, Qiang Cheng, Justin F. Fraser, Liyu Gong, Lisa A. Collier, Stephanie M. Davis, Doug Lukins, Abdulnasser Alhajeri, Stephen Grupke, Keith R. Pennypacker

**Affiliations:** ^1^School of Nursing, University of Washington, Seattle, WA, United States; ^2^Institute for Biomedical Informatics, University of Kentucky, Lexington, KY, United States; ^3^Department of Neurology, University of Kentucky, Lexington, KY, United States; ^4^College of Medicine, University of Kentucky, Lexington, KY, United States; ^5^Departments of Neurosurgery, University of Kentucky, Lexington, KY, United States; ^6^Neuroscience, University of Kentucky, Lexington, KY, United States; ^7^Radiology, University of Kentucky, Lexington, KY, United States

**Keywords:** ischemic stroke, machine learning, gene expression, cytokines, chemokines

## Abstract

**Introduction:** Ischemic stroke remains one of the most debilitating diseases and is the fifth leading cause of death in the US. The ability to predict stroke outcomes within the acute period of stroke would be essential for care planning and rehabilitation. The Blood and Clot Thrombectomy Registry and Collaboration (BACTRAC; clinicaltrials.gov NCT03153683) study collects arterial blood immediately distal and proximal to the intracranial thrombus at the time of mechanical thrombectomy. These blood samples are an innovative resource in evaluating acute gene expression changes at the time of ischemic stroke. The purpose of this study was to identify inflammatory genes and important immune factors during mechanical thrombectomy for emergent large vessel occlusion (ELVO) and which patient demographics were predictors for stroke outcomes (infarct and/or edema volume) in acute ischemic stroke patients.

**Methods:** The BACTRAC study is a non-probability sampling of male and female subjects (≥18 year old) treated with mechanical thrombectomy for ELVO. We evaluated 28 subjects (66 ± 15.48 years) relative concentrations of mRNA for gene expression in 84 inflammatory molecules in arterial blood distal and proximal to the intracranial thrombus who underwent thrombectomy. We used the machine learning method, Random Forest to predict which inflammatory genes and patient demographics were important features for infarct and edema volumes. To validate the overlapping genes with outcomes, we perform ordinary least squares regression analysis.

**Results:** Machine learning analyses demonstrated that the genes and subject factors CCR4, IFNA2, IL-9, CXCL3, Age, T2DM, IL-7, CCL4, BMI, IL-5, CCR3, TNFα, and IL-27 predicted infarct volume. The genes and subject factor IFNA2, IL-5, CCL11, IL-17C, CCR4, IL-9, IL-7, CCR3, IL-27, T2DM, and CSF2 predicted edema volume. The overlap of genes CCR4, IFNA2, IL-9, IL-7, IL-5, CCR3, and IL-27 with T2DM predicted both infarct and edema volumes. These genes relate to a microenvironment for chemoattraction and proliferation of autoimmune cells, particularly Th2 cells and neutrophils.

**Conclusions:** Machine learning algorithms can be employed to develop prognostic predictive biomarkers for stroke outcomes in ischemic stroke patients, particularly in regard to identifying acute gene expression changes that occur during stroke.

## Introduction

Ischemic stroke is the fifth cause of death in the United States, accounting for 87% of all strokes (1). Approximately 800,000 individuals are affected per year and ischemic stroke is the primary cause of severe long-term disability (1). When a major cerebral artery is blocked by a thrombus, the occlusion causes deprivation of oxygen, glucose, and other essential nutrients to the brain which results in necrotic cerebral tissue (2). Current treatments focused on recanalization of the occluded cerebral artery for emergent large vessel occlusions (ELVO) via intravenous administration of tissue plasminogen activator (tPA) and/or endovascular mechanical thrombectomy (3–5). Stroke patients must be given tPA within a narrow window of 4.5 h, while mechanical thrombectomy has an extended window of intervention up to 24 h post-stroke (6–8). However, ischemic stroke patients must meet particular imaging or time criteria to receive either or both treatments, and many patients are out of the “window of opportunity,” and receive only supportive medical care (9).

With early reperfusion to the cerebral artery, at risk tissue in the penumbra may be salvaged (10, 11). Even if blood flow is restored, neurons in the penumbra face major challenges to their survival, such as acidosis, ion pump failure, excitotoxicity, inflammatory response, and the breakdown the blood brain barrier (BBB) (12–14). Inflammation following an acute ischemic stroke is multifaceted, and is part of the normal pathological processes involved in the post-ischemic brain. Within the first few hours, there is a rapid activation of resident microglia that start to release proinflammatory mediators from the ischemic endothelium and brain parenchyma [including interleukin-(IL)1β, IL-6, IL-12, IL-23, and tumor necrosis factor-α (TNF-α)] (15–17). Furthermore, there is an activation of peripheral immune cells including neutrophils and T-cells that can cross the damaged BBB and accumulate at the injury site in the microvascular environment. In this acute phase of ischemic stroke more extensive damage occurs with the associated increased intracranial pressure leading to malignant cerebral edema, neuronal death, hemorrhagic transformation, and mortality (18–21).

Mechanical thrombectomy has greatly improved the ability to restore blood flow and improve patient outcomes, even when used in selected patients up to 24 h post-stroke onset (5, 22). The process of thrombectomy permits us to extract blood directly in the vicinity of the infarct from a stroke patient to provide the first glimpse into cellular/molecular events occurring during an ischemic stroke. Through the standard thrombectomy process, we can isolate distal blood within the artery immediately downstream from the clot, peripheral blood that is proximal to the clot (systemic arterial blood in the cervical carotid artery), and the thrombus itself upon removal from the human subject (23).

Understanding of molecular and cellular changes intraluminally in ischemic stroke may provide develop deeper insights into the inflammatory pathways involved, and could lead to the identification of prognostic biomarkers in stroke patients. This information could potentially provide benchmarks to study responses to treatment and aid in development of new therapies. The purpose of this study was to identify inflammatory genes and important immune mediators at the time of mechanical thrombectomy and which patient demographics were predictors for stroke outcomes (infarct and/or edema volume) in acute ischemic stroke patients.

## Methods

### Study Design

This study was developed from the Blood and Clot Thrombectomy Registry and Collaboration (BACTRAC) protocol: a prospectively enrolling tissue bank for emergent large vessel occlusion (ELVO) (clinicaltrials.gov NCT03153683) (23). The BACTRAC study is a non-probability sampling of subjects over 18 years old, conducted at a large academic comprehensive stroke center in central Kentucky, and who were treated with mechanical thrombectomy for ELVO ischemic stroke. While the BACTRAC tissue bank is continuously enrolling, data for this study was collected from subjects enrolled May 2017 through January 2019. The Institutional Review Board approved this study, and informed consent was obtained from all subjects or legally authorized representatives.

### Sample Population

Subjects (≥18 years old) were eligible for inclusion if they were hospitalized with a diagnosis for an ischemic stroke and underwent thrombectomy procedure for ELVO. Subjects were candidates for inclusion if they met criteria: informed consent was signed and dated by subject or legally authorized representative within 24 h post-thrombectomy, confirmation of acute ischemic stroke based on clinical and radiographic imaging (CT and/or MRI), intra-arterial thrombectomy as determined by neurointerventional radiology (NIR) physician, an acute thromboembolus within an intracranial artery and underwent endovascular thrombectomy procedure. Subjects were excluded if baseline urine pregnancy test was positive and/or breastfeeding and/or concurrently participated in treatment studies.

### Data Collection

A medical record review was conducted to collect admission clinical and demographic variables. These variables included age, sex, ethnicity, height, weight, body mass index (BMI), comorbidities, smoking status, National Institutes of Health Stroke Scale (NIHSS), baseline computerized tomography (CT) and/or magnetic resonance imaging (MRI) scan to verify large vessel occlusion stroke/thrombectomy eligibility and CTA collateral score, and time of last known normal (LKN) symptom (prior to ischemic stroke).

Tissue removal and tissue banking protocols were previously published in detail (23). In brief, this was a collaborative effort among the NeuroInterventional Radiology (NIR) service line and our Center for Advanced Translational Stroke Science. At the beginning of the mechanical thrombectomy procedure, during initial access through the clot to the distal intracranial vessels, the NIR physician backbleeds the microcatheter to determine if they are distal to the distal end of the thrombus. Through this, 1 ml of whole arterial blood from the microcatheter (0.021 inch inner lumen diameter) was collected. Simultaneously, the collection of 7 ml of whole arterial peripheral blood was collected from the cervical parent artery (internal carotid artery or vertebral artery) during backbleeding while initially suction for thrombectomy. The distal (intracranial) and proximal (peripheral) blood samples were handed off to the on-call research associates who immediately processed the specimens in the wet laboratory adjacent to the angiography suite.

An additional medical chart review was conducted to gather post-thrombectomy endpoint variables: last known normal (LKN) symptom to thrombectomy completion time (infarct time), thrombolysis in cerebral infarction (TICI) scores, and NIHSS at discharge. A blinded neuroradiologist evaluated post-thrombectomy computed tomography (CT) imaging and/or magnetic resonance imaging (MRI) for infarct and edema volumes, and hemorrhagic grade. Clinical and demographic variables, along with post-thrombectomy endpoints were deidentified and maintained in our institution's REDCap software.

### RNA Extraction and Amplification

The extraction of total RNA from the cellular pellet/buffy coat was collected from the stored distal and proximal arterial blood samples using the Nucleospin Blood Kit (Macherey-Nagel, Düren, Germany), per manufacturer protocol. Nanodrop Lite Spectrophotometer (Thermo-Fisher Scientific; Waltham, MA) estimated the quantity of total RNA based on the A260/A280 ratio. To validate total RNA, RNA integrity numbers (RIN) values were obtained from the hospital's Genomics Core facility. The assessment and analysis of RNA integrity was evaluated with Eukaryote Total RNA Nano assay on the Agilent 2100 Bioanalyzer (Agilent Technologies, Santa Clara, CA) and with associated Agilent 2100 Bioanalyzer expert software. The mean RIN values (±SD) for distal and proximal arterial blood samples were 8.89 ± 0.41 (range from 8.30 to 9.60) and 6.98 ± 0.29 (range from 6.60 to 7.50), respectfully. The RIN values indicate moderate to high quality RNA with minimal degradation (24). RT^2^ First Strand Kit (Qiagen) was used to synthesize cDNA which were analyzed for gene expression using Qiagen RT^2^ Profiler PCR Array Human Inflammatory Cytokines & Receptors for 84 genes associated with inflammation and evaluated with StepOnePlus Real-Time PCR System interfaced with StepOne software (Applied Biosystems, Foster City, CA). Each gene array plate had five housekeeping genes (ACTB, B2M, GAPDH, HPRT1, and RPLP0) to normalize the gene expression data by using ΔΔCT method. The distal blood data for each gene was compared to proximal blood data for each subject. Proximal blood samples were used as an internal control for each patient, since there is not a true control. Since the cervical segments have circulating blood, we believe these are equivalent to arterial blood taken from peripheral arterial location.

### Data Analysis

Descriptive and frequency statistics were obtained and used to characterize male and female ischemic stroke subjects. Comparisons were made for subject demographics and baseline characteristics using SPSS, version 26 software (IBM, Armonk, NY). Statistical significance (*p*-value of 0.05) for group differences were assessed by independent *t*-tests for continuous variables and Chi-square or Fisher exact tests of associations based on categorical (ordinal and binary) variables. The gene expression mean fold change values were assessed with Scikit-learn Python package (v0.21.2) for machine learning analysis (25). Statesmodels Python package (v0.10.1) was used for ordinary least squares (OLS) regression analysis (26).

### Machine Learning

For machine learning analysis, the demographics data used included variables: sex, age, race/ethnicity, BMI, comorbidities (hypertension, type 2 diabetes, hyperlipidemia, previous stroke, and atrial fibrillation), and two stroke outcomes (infarct and edema volumes). The gene expression dataset included 84 inflammatory genes. Any variables in the two stroke outcome datasets were dropped if the proportion of missing values were more than 40%. After pre-processing 89 variables were in the final dataset.

### Predicting Infarct Volume

Predictive analysis was performed by using the infarct volume as the response variable, and all gene expression variables as predictors. The analysis was adjusted by using demographic variables as covariates. Regression was used to perform the predictive analysis task. Since there was a relatively small sample size with a mild number of predictors (i.e., 28 subjects with 89 predictors), a widely used machine learning method, Random Forest (RF) (27, 28) was employed to complete the analysis. This method was used because of the reliable performance on small sample datasets with mixed categorical and continuous variables.

RF is an ensemble of decision trees, where each internal node represents a condition, or test, on a single predictor, aiming to split the dataset into two so that each had a similar response values. RF provides versatile, accurate, and stable predictions for supervised machine learning tasks such as regression as well as measuring the relative importance of each predictor. The importance score of a predictor is calculated by how much the variance is reduced or information gain is increased across all nodes that were used. The importance scores were scaled so the sum equals one.

Hyper-parameters of RF were selected in a coarse-to-fine approach using *k*-fold cross-validation (CV), which partitions the training dataset into *k* parts evenly, then rotationally uses *k-1* parts to train the machine learning model and tests the model with the remaining one part. The parameters were first selected using the 5-fold CV, then tuned the parameters around the selected value using the 10-fold CV. Predicting performance of RF was optimized in mean squared error (MSE), which is minimized for the optimal model.

With the selected hyper-parameters, the importance of features using RF were ranked. Ranking may be affected by randomness of RF due to the correlations between some features. Therefore, we programed RF 100 times and collected the mean values of the feature importance. The number of possible orders of the features is combinatorically large, this approach can effectively reduce but cannot fully eliminate the effect of the correlation between features.

### Predicting Edema Volume

Similar to the prediction of infarct volumes, this was a regression task by using edema volumes as a response variable and the remaining gene variables as predictors. The analysis was adjusted using the demographic variables as covariates as done for the analysis of infarct volumes. Using similar preprocessing and cross validation with RF, the negative MSE was used as a score and maximized for optimal RF model. The relative importance of the predictors were also obtained for this task.

### Stroke Outcomes of Infarct and Edema Volumes

Noncontrast head CT and CTA of the head and neck were obtained upon presentation to the emergency department during initial assessment for acute ischemic stroke. Siemens SOMATOM Definition Edge and SOMATOM Force CT scanners were used for all CT studies. CTA collateral scores were determined using maximum intensity projection images from CTA of the head with a scoring system described in previous work by Souza et al. (29). MRI and CT of the head without contrast were obtained following thrombectomy. MRI was performed using Siemens MAGNETOM Aera and MAGNETOM Skyra machines at magnetic field strength of 1.5 and 3.0 Tesla, respectively. Hemorrhage grade, infarct volume, and edema volume were determined on post-thrombectomy MRI of the head or CT of the head if MRI was unavailable. Hemorrhage grade was determined using a grading scale described by Hacke et al. (30). Infarct volumes and edema volumes were calculated using post-thrombectomy MRI, or using CT if MRI was unavailable. Imaging included the entire brain on both MRI and CT examinations, and all images/slices were visually assessed for infarction or edema. When MRI was available, diffusion weighted images (DWI) were used to calculate infarct volumes, and T2 FLAIR images were used to calculate edema volumes. The areas of abnormal signal (restricted diffusion on DWI or hyperintense signal on T2 FLAIR) were manually segmented and analyzed to determine volume using ITK-SNAP software (www.itksnap.org) (31). When MRI was unavailable, CT of the head was used to calculate both infarct and edema volume. As infarct and edema were indistinguishable on CT, the same value was used for both after manually segmenting the area of abnormal hypoattenuation using ITK-SNAP. All imaging assessment, including hemorrhage grade, CTA collateral score, infarct volume, and edema volume was performed by an experienced neuroradiologist in a blinded fashion.

## Results

### Sample Characteristics

Twenty-eight subjects underwent mechanical thrombectomy during the study period. The mean age of our subjects were 66 ± 15.48 years, primarily female (61%), and the majority were Caucasian (86%). Table 1 presents baseline subject characteristics and comparisons based on sex. Similarities were exhibited between the subjects for BMI, comorbidities (hypertension, atrial fibrillation, type 2 diabetes, hyperlipidemia, previous stroke, chronic obstructive pulmonary disease, and coronary artery disease), smoking status, stroke location, hemorrhagic grade, CTA collateral scores, infarct and edema volumes. The NIHSS on average went from admission score of 17 ± 6.56 to discharge score of 11 ± 9.54 post-thrombectomy. More males (81.8%) than females (41.2%) exhibited full perfusion to the occluded artery post-thrombectomy with a TICI score of 3 (*p* = 0.034). The mean infarct time (LKN to thrombectomy completion time) for males was ~7 h vs. almost 10 h for females (*p* = 0.093).

**Table 1:** Baseline demographics and characteristics for ischemic stroke subjects.

	**Males (*n* = 11)**	**Females (*n* = 17)**	***p*-value**
**Age (years)**	68 ± 17.07	64 ± 14.77	0.606
**Ethnicity**			
African-American	2 (18.2)	0 (0.0)	0.072
Caucasian	9 (81.8)	15 (88.2)	0.650
Unknown	0 (0.0)	2 (11.8)	0.254
**BMI**			
Under/normal weight	4 (36.4)	9 (52.9)	0.409
Overweight	6 (54.5)	6 (35.3)	0.333
Obese	0 (0.0)	0 (0.0)	–
Morbidly obese	1 (9.1)	2 (11.8)	0.831
**Comorbidities**			
Hypertension	4 (36.4)	11 (64.7)	0.346
Atrial fibrillation	3 (27.3)	5 (29.4)	0.907
Diabetes	1 (9.1)	6 (35.3)	0.127
Hyperlipidemia	3 (27.3)	2 (11.8)	0.313
Previous stroke	2 (18.2)	3 (17.6)	0.973
COPD	1 (9.1)	3 (17.6)	0.545
CAD	3 (27.3)	1 (5.9)	0.123
**Smoking status**			
Never	5 (45.5)	11 (64.7)	0.333
Currently	4 (36.4)	4 (23.5)	0.481
Previously (>6 months)	2 (18.2)	2 (11.8)	0.650
**NIHSS on admission**	17 ± 8.04	16 ± 5.65	0.732
Minor stroke (1-4)	2 (18.2)	1 (5.9)	0.322
Moderate stroke (5-15)	3 (27.3)	6 (35.3)	0.671
Moderate/severe (16-20)	2 (18.2)	6 (35.3)	0.346
Severe stroke (≥21)	4 (36.4)	4 (23.5)	0.481
**NIHSS at discharge**	14 ± 14.21	9 ± 7.32	0.249
Minor stroke (1-4)	4 (36.4)	6 (35.3)	0.808
Moderate stroke (5-15)	4 (36.4)	7 (41.2)	0.818
Moderate/severe (16-20)	1 (9.1)	2 (11.8)	0.838
Severe stroke (≥21)	2 (18.2)	1 (5.9)	0.322
**TICI score**			
2A ≤ 50% Perfusion	0 (0.0)	1 (5.9)	0.432
2B ≥ 50% Perfusion	2 (18.2)	9 (52.9)	0.070
3 = Full perfusion	9 (81.8)	7 (41.2)	0.034
**LKN to thrombectomy completion time (minutes)**	416 ± 231.88	582 ± 249.06	0.093
**Stroke location**			
Left MCA	4 (36.4)	8 (47.1)	0.576
Right MCA	6 (54.5)	8 (47.1)	0.699
Basilar	1 (9.1)	1 (5.9)	0.747
**Infarct volume (cm**^**3**^**)**	87.90 ± 104.58	48.33 ± 62.30	0.219
**Edema volume (cm**^**3**^**)**	98.60 ± 116.01	48.52 ± 61.23	0.147
**Hemorrhagic grade**			
None	4 (36.4)	4 (23.5)	0.481
HI1	4 (36.4)	7 (41.2)	0.808
H12	1 (9.1)	6 (35.3)	0.127
PH1	1 (9.1)	0 (0.0)	0.220
PH2	1 (9.1)	0 (0.0)	0.220
**CTA collateral score**			
0	3 (27.3)	2 (11.8)	0.650
1	5 (45.5)	11 (64.7)	0.333
2	3 (27.3)	4 (23.5)	0.748

*Values are mean ± SD or (%). Comparisons were performed with independent t-tests, Chi-square, or Fisher exact tests based on distribution of data*.

### Prediction of Infarct Volume With Machine Learning

For predicting infarct volume, the covariates including all 84 genes and the 10 demographic variables are all ranked in importance scores. For the ranked top *k, k* = *1–84*, covariates we perform 5-fold CVs to find the optimal RF model and obtain the corresponding optimal performance, which is scored in negative MSE. The importance values of the top 10 covariates, 10 for predictive analysis of infarct volume are given in Table 2. They are in descending order as follows: CCR4, IFNA2, IL-9, CXCL3, age, type 2 diabetes (T2DM), IL-7, CCL4, BMI, IL-5, CCR3, TNFα, and IL-27.

**Table 2:** Importance values of top 10 variables for predicting infarct volume.

CCR4	0.077
IFNA2	0.063
IL-9	0.053
CXCL3	0.045
Age	0.038
Type 2 diabetes	0.036
IL-7	0.034
CCL4	0.029
BMI	0.028
IL-5	0.027
CCR3	0.023
TNFα	0.022
IL-27	0.021

### Prediction of Edema Volume With Machine Learning

For predictive analysis of edema volume, in a similar way to the predictive analysis of infarct volume, we obtain the optimal performance for the top *k* covariates, *k* = 1–84. The importance values of the top 10 most important covariates are shown in Table 3. They are in descending order as follows: IFNA2, IL-5, CCL11, IL-17C, CCR4, IL-9, IL-7, CCR3, IL-27, T2DM, and CSF2.

**Table 3:** Importance values of top 10 variables for predicting edema volume.

IFNA2	0.074
IL-5	0.070
CCL11	0.057
IL-17C	0.045
CCR4	0.045
IL9	0.044
IL-7	0.036
CCR3	0.035
IL-27	0.033
Type 2 diabetes	0.032
CSF2	0.025

When using infarct and edema volumes as outcome variables, we observed selected gene subsets for these two predictive analysis have a significant overlap. The intersection of the top 10 genes from the two stroke outcomes are seven genes with T2DM, these include CCR4, IFNA2, IL-9, IL-7, IL-5, CCR3, and IL-27. This overlapping subset of genes were regarded as the most important associations at the time of mechanical thrombectomy.

### Prediction of Stroke Outcomes With Multiple Linear Regression

To validate this overlapping subset of genes, we perform ordinary least squares (OLS) regression analysis with the top seven covariates. The OLS results for predictors of infarct volume and edema volume are shown in Tables 4, 5, respectively. To note, the OLS was a statistical model that assumes a linear relationship between the predictive outcome and the covariates. In contrast, the machine learning model was capable of capturing potentially highly non-linear relationship between the outcome and the covariates (32, 33). Therefore, OLS may partially capture the association between the outcomes and the covariates. IL-9 and T2DM were predictors for infarct volume [F(8,27 = 5.396, *p* = 0.001). These predictors for infarct volume explained 53% of the total variance in the model (Table 4). Similarly, we performed the OLS analysis for predictors of edema volume. IL-9 was a predictor for edema volume [F(8,27 = 2.938, *p* = 0.028). IL-9 explains 33% of the total variance found in the model (Table 5).

**Table 4:** OLS regression variables predicting infarct volume (*n* = 28).

**Variable**	**β**	***P*-value**
CCR4	−0.733	0.740
IFNa2	−0.104	0.793
IL-9	12.146	0.017
Type 2 diabetes	69.942	0.026
IL-7	−5.277	0.217
IL-5	1.205	0.233
CCR3	−0.084	0.855
IL-27	−0.683	0.549

*R^2^ = 0.654, adjusted R^2^ = 0.533, df = 8, F = 5.396, p = 0.001*.

*Durbin-Watson = 1.83*.

**Table 5:** OLS regression variables predicting edema volume (*n* = 28).

**Variable**	**β**	***P*-value**
CCR4	−1.987	0.486
IFNa2	−0.099	0.848
IL-9	13.127	0.040
Type 2 Diabetes	57.469	0.140
IL-7	−8.326	0.134
IL-5	2.109	0.110
CCR3	0.083	0.889
IL-27	−0.729	0.693

*R^2^ = 0.507, adjusted R^2^ = 0.334, df = 8, F = 2.938, p = 0.028*.

*Durbin-Watson = 1.69*.

### Gene Expression of Inflammatory Markers and Ingenuity Pathways Analysis

Ten inflammatory genes (CCR4, IFNA2, IL-9, CXCL3, IL-7, CCL4, IL-5, CCR3, TNFα, and IL-27) were recognized as predictors for infarct volume had mean fold change ranging from 2.56 to 35.06. While the inflammatory genes (IFNA2, IL-5, CCL11, IL-17C, CCR4, IL-9, IL-7, CCR3, IL-27, and CSF2) that were identified as predictors for edema volume had mean fold change ranging from 3.98 to 42.61. The seven important predictor genes (CCR4, IFNA2, IL-9, IL-7, IL-5, CCR3, and IL-27) that overlapped both stroke outcomes had mean fold change ranging from 3.98 to 35.06.

**Figure 1:**
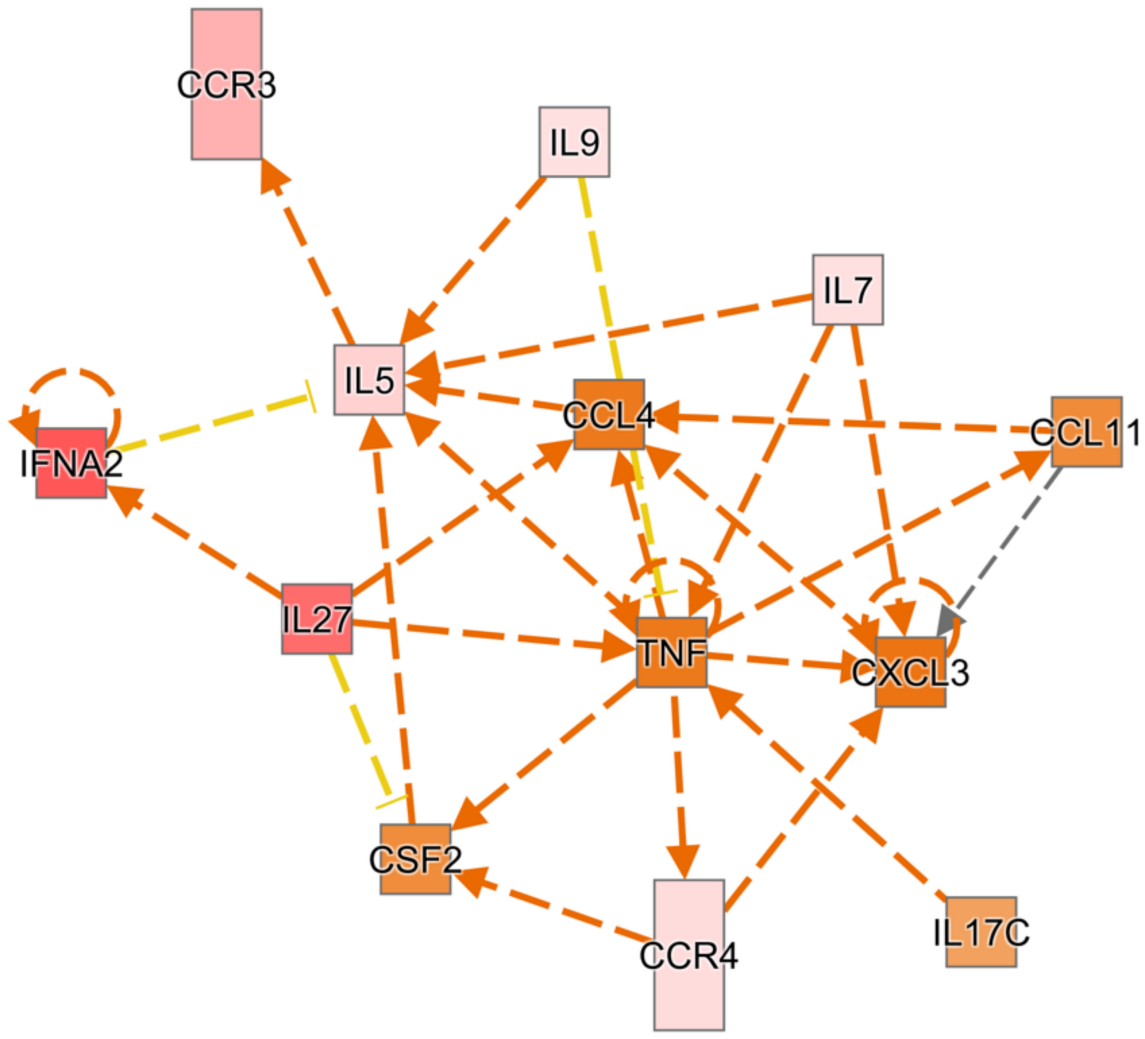
Ingenuity pathway analysis predicts the upstream and downstream effects of activation or inhibition of the 13 genes from the distal blood in a network. A red box indicates the gene is more extreme in the dataset, while different shades of pink mean the gene is measured less in the dataset. An orange box indicates the gene has more confidence in predicted activation. Directional orange arrows indicate which gene leads to activation of another gene. Yellow arrows indicate the findings are inconsistent with downstream gene.

The mean fold changes for the 13 genes were analyzed through the use of Ingenuity Pathway Analysis (IPA) software (Qiagen Inc., https://www.qiagenbioinformatics.com/products/ingenuity-pathway-analysis) (34). IPA predicts the upstream and downstream effects of activation or inhibition of the 13 genes from the distal blood in a pathway network. The overall inflammatory chemokine/cytokine pattern from the distal blood characterizes a microenvironment for chemoattraction and proliferation of immune cells, particularly in Th2 response and neutrophils (Figure 1).

## Discussion

Our results revealed insights to the initial inflammatory response to ischemic stroke in stroke patients. Arterial blood samples were collected at one of the earliest timepoints (~8 h and 35 min from LKN to thrombectomy completion time or infarct time) during an ongoing stroke. Expression of 13 cytokines and chemokines were induced over 2-fold in the distal blood relative to levels in the proximal blood. Machine learning identified 10 genes, along with age, T2DM, and BMI for predictors of infarct volume and 10 genes with T2DM for predictors of edema volume. In addition, there was an overlap with seven genes and T2DM as predictors for both stroke outcomes.

These seven genes are related to the Th2 response, which is a T-cell response associated with allergy, cancer, and autoimmunity (35, 36). One study has shown a systemic shift of the immune system toward Th2 response at the late post-acute phase in stroke patients after examining their peripheral venous blood (37). IFNA2 is primarily produced by dendritic cells (38). This cytokine is targeted as a treatment for the autoimmune disease, systemic lupus erythematosus (39). Exposure to INFA2 has been linked to causing edema and been shown to induce the production of cytokine IL-7 (40–42). IL-7 is required for T-cell development and function (43, 44). The expression of IL-4, a classic marker for Th2 cells, is increased by IL-7 and is a direct cause of edema (45, 46). This cytokine's activity is targeted for diseases related to pathogenic T-cells (47). Th2 cells express both CCR3 and CCR4 receptors that bind chemokines to direct their migration to the site of injury or infection (48, 49). CCR3 is a chemokine receptor that has been shown to mediate neuronal injury after ischemic insult (50). At least eight different chemokines recognize this receptor and has shown that its activation enhances choroidal neovascularization and trafficking of eosinophils (51). Pharmaceutical companies have targeted this receptor for the treatment of severe asthma (52). CCR4 is another chemokine receptor and CCL17 and CCL22 are identified as high affinity ligands (53). This receptor is highly expressed in thymus and peripheral blood leukocytes, and is targeted for treatment in T-cell leukemia and peripheral T-cell lymphoma (54). The cytokines, IL-5 and IL-9 are both expressed and secreted by Th2 cells and are linked to edema and infarct volume, respectively (55, 56). IL-5 is produced in female mice express higher levels in the spleen relative to males (57). Antagonists to IL-5 have been produced for the treatment of severe asthma (58). Patients suffering from asthma have higher levels of IL-9 producing T-cells in their peripheral blood (59). While not targeted in a current treatment, systemically applied neutralizing antibodies reduces infarct (56). Cytokine, IL-27 has both pro-and anti-inflammatory effects and the main source of activation is by antigen-presenting cells (60). IL-27 activates an inflammatory response by inducing CD4^+^ T-cells into a Th1 response (61–64). However, IL-27 can also suppress Th17 differentiation and inhibit inflammation enhancing a Th2 response (65–68). In an experimental stroke model, mice were administered IL-27 injections post-intracranial hemorrhage (ICH) and demonstrated improved behavior tests, mobility, and reduced edema around the hemorrhage sites (69).

Several limitations to this study should be noted. The sample for this study was small and subjects were mostly Caucasian due to hospital location. There was a potential for sampling bias since this was a non-probability strategy and based on completed mechanical thrombectomy procedures. A more diverse and larger sample size is warranted in future studies. Standard of care imaging for infarct and edema volumes were based on CT and/or MRI imaging post-thrombectomy. Additional imaging at discharge for comparison is warranted to understand how thrombectomy relates to these stroke outcomes. Follow-up assessment would be helpful to address subject's long-term recovery and functional status.

## Conclusion

The machine learning (ML) algorithm has uncovered a molecular/cellular signaling pathway that the literature supports its relationship with the facilitation of infarct volume and edema. Despite significant funding for stroke drug discovery, tPA and mechanical thrombectomy are the only treatments. Identifying the initial activators of the inflammatory response to stroke could be therapeutic targets for treatment. The inhibition of these early inflammatory events has the potential to block the inflammatory cascade that occurs after a stroke that recruits the peripheral immune system, which is involved in neurodegeneration that lasts days afterward. The ML method has determined several related genes are predictors of edema and infarct volume in acute ischemic stroke patients. Thus, the use of ML to determine molecular predictors from ischemic blood could initiate a new approach for drug discovery in the field of stroke. Future studies will utilize ML methods and examine expression of genes that predict long-term functional outcomes.

## Data Availability Statement

The datasets generated for this study are available on request to the corresponding author.

## Ethics Statement

The studies involving human participants were reviewed and approved by UK HealthCare Office of Research Integrity IRB # 17-0019-F1V clinicaltrials.gov NCT03153683. The patients/participants provided their written informed consent to participate in this study.

## Author Contributions

SM, JF, and KP: conceptualization, review, and editing. SM, LC, SD, QC, LG, DL, AA, SG, JF, and KP: methodology. QC and LG: formal analysis. SM, QC, LG, DL, and KP: written original draft. All authors made a substantial and intellectual contribution to the work.

### Conflict of Interest

The authors declare that the research was conducted in the absence of any commercial or financial relationships that could be construed as a potential conflict of interest.
